# Feasibility and Safety of Tailored Lymphadenectomy Using Sentinel Node-Navigated Surgery in Patients with High-Risk T1 Esophageal Adenocarcinoma

**DOI:** 10.1245/s10434-023-13317-6

**Published:** 2023-03-23

**Authors:** Charlotte N. Frederiks, Anouk Overwater, Jacques J. G. H. M. Bergman, Roos E. Pouw, Bart de Keizer, Roel J. Bennink, Lodewijk A. A. Brosens, Sybren L. Meijer, Richard van Hillegersberg, Mark I. van Berge Henegouwen, Jelle P. Ruurda, Suzanne S. Gisbertz, Bas L. A. M. Weusten

**Affiliations:** 1 Department of Gastroenterology and Hepatology, St. Antonius Hospital, Nieuwegein, The Netherlands; 2 Department of Gastroenterology and Hepatology, University Medical Center Utrecht, Utrecht University, Utrecht, The Netherlands; 3 Department of Gastroenterology and Hepatology, Amsterdam University Medical Centers, Amsterdam, The Netherlands; 4 Department of Radiology and Nuclear Medicine, University Medical Center Utrecht, Utrecht University, Utrecht, The Netherlands; 5 Department of Radiology and Nuclear Medicine, Amsterdam University Medical Centers, Amsterdam, The Netherlands; 6 Department of Pathology, University Medical Center Utrecht, Utrecht University, Utrecht, The Netherlands; 7 Department of Pathology, Amsterdam University Medical Centers, Amsterdam, The Netherlands; 8 Department of Surgery, University Medical Center Utrecht, Utrecht University, Utrecht, The Netherlands; 9 Department of Surgery, Amsterdam University Medical Centers, Amsterdam, The Netherlands; 10 Cancer Treatment and Quality of Life, Cancer Center Amsterdam, Amsterdam, The Netherlands

## Abstract

**Background:**

Selective lymphadenectomy using sentinel node-navigated surgery (SNNS) might offer a less invasive alternative to esophagectomy in patients with high-risk T1 esophageal adenocarcinoma (EAC). The aim of this study was to evaluate the feasibility and safety of a new treatment strategy, consisting of radical endoscopic resection of the tumor followed by SNNS.

**Methods:**

In this multicenter pilot study, ten patients with a radically resected high-risk pT1cN0 EAC underwent SNNS. A hybrid tracer of technetium-99m nanocolloid and indocyanine green was injected endoscopically around the resection scar the day before surgery, followed by preoperative imaging. During surgery, sentinel nodes (SNs) were identified using a thoracolaparoscopic gammaprobe and fluorescence-based detection, and subsequently resected. Endpoints were surgical morbidity and number of detected and resected (tumor-positive) SNs.

**Results:**

Localization and dissection of SNs was feasible in all ten patients (median 3 SNs per patient, range 1–6). The concordance between preoperative imaging and intraoperative detection was high. In one patient (10%), dissection was considered incomplete after two SNs were not identified intraoperatively. Additional peritumoral SNs were resected in four patients (40%) after fluorescence-based detection. In two patients (20%), a (micro)metastasis was found in one of the resected SNs. One patient experienced neuropathic thoracic pain related to surgery, while none of the patients developed functional gastroesophageal disorders.

**Conclusions:**

SNNS appears to be a feasible and safe instrument to tailor lymphadenectomy in patients with high-risk T1 EAC. Future research with long-term follow-up is warranted to determine whether this esophageal preserving strategy is justified for high-risk T1 EAC.

**Supplementary Information:**

The online version contains supplementary material available at 10.1245/s10434-023-13317-6.

Endoscopic resection is established as the first-choice treatment for early esophageal adenocarcinoma (EAC) limited to the (sub)mucosa. Depending on the presence of histopathologic features associated with lymph nodes metastases (LNM), additional surgery might be indicated. For radically resected low-risk cancers [i.e., mucosal or submucosal invasion < 500 μm, good/moderate differentiation, and absence of lymphovascular invasion (LVI)], endoscopic resection is considered to be curative since the risk of lymphatic spread of tumor cells to adjacent lymph nodes is negligible (< 2%).^1–4^ However, the risk of concomitant LNM is higher when one or more histopathologic risk factors (i.e., submucosal invasion > 500 μm, poor differentiation, and LVI) are present in the endoscopic resection specimen. Although the reported incidence rates of LNM vary from 0 to 20% in high-risk mucosal (T1a) EAC,^3,5^ there is no consensus on the optimal management for this subcategory, which drives heterogeneous clinical decision making. For submucosal (T1b) cancer with high-risk features, current guidelines recommend esophagectomy on the basis of reported LNM incidence rates of up to 37%.^2–7^

However, esophagectomy is a major surgical procedure associated with significant morbidity (up to 65%), mortality (0–5%), and reduced quality of life postoperatively.^8–12^ Considering early T1 EAC can often be radically removed with endoscopic resection, surgical resection of the esophagus and all locoregional lymph nodes is only performed in view of the risk of LNM. However, the relatively low risk of LNM questions the need for immediate adjuvant surgery in a large subset of patients with high-risk T1 EAC.

A less invasive alternative might be a selective lymphadenectomy using sentinel node-navigated surgery (SNNS), after which additional treatment can be tailored based on lymph node involvement. As compared with esophagectomy, this alternative approach would preserve the upper gastrointestinal anatomy, and thereby possibly reduce the risk of postoperative complications and decreased quality of life. The concept of SNNS is already widely used to personalize lymph node dissection in other malignancies, while the number of studies in EAC is still limited.^13^ Thus far, early T1 tumors are considered the most suitable candidates, since failure of sentinel node (SN) mapping is frequently reported in advanced carcinomas due to lymph vessel destruction by the tumor and/or neoadjuvant therapy.^14^

Based on these ideas, a recent study from our group found SNNS using a radioactive tracer technetium-99m (^99m^Tc) nanocolloid to be feasible and safe in patients with high-risk T1 EAC. However, one tumor-positive SN located in the peritumoral region was missed as a result of high tracer activity at the injection site, known as the shine-through effect.^15^ Following these findings, the tracer was enhanced by adding indocyanine green (ICG), which can be visualized during surgery with near-infrared (NIR) light. Recently, we showed that this combination of ICG with ^99m^Tc nanocolloid improves the detection and visualization of peritumoral SNs.^16^

Whereas in these preceding studies SNNS was always followed by an esophagectomy in the same session, the esophagus is not resected in the current study. This is therefore the first study to investigate the feasibility and safety of a new treatment algorithm for patients with high-risk T1 EAC, consisting of a radical endoscopic resection of the tumor followed by tailored lymphadenectomy using SNNS with a hybrid tracer of ^99m^Tc nanocolloid and ICG. Here we report the short-term outcomes on this esophageal preserving treatment regimen, which might be a valuable option in carefully selected patients.

## Patients and Methods

### Study Design and Patient Population

For this prospective multicenter pilot study, patients were included in two tertiary hospitals in the Netherlands [Amsterdam University Medical Centers, Amsterdam, and University Medical Center Utrecht, Utrecht]. Patients were eligible after a radical endoscopic resection of a high-risk T1 EAC without the clinical presence of lymph node or distant metastases (i.e., cN0M0) as determined by preoperative staging with PET–CT and endoscopic ultrasound. Two histolopathological subgroups were distinguished: (1) high-risk T1a EAC, defined as intramucosal cancer, with poor differentiation (G3/4), and/or LVI, and (2) high-risk T1b EAC, defined as submucosal cancer with deep invasion in the submucosa (≥ 500 μm, SM2/3), and/or poor differentiation, and/or LVI. Known allergy for ^99m^Tc nanocolloid or ICG, prior neoadjuvant (chemo)radiation therapy, previous surgery or comorbidity interfering with the study procedures, and another primary tumor with a life-expectancy ≤ 3 years were exclusion criteria.

### Sentinel Node-Navigated Surgery

**Fig. 1:**
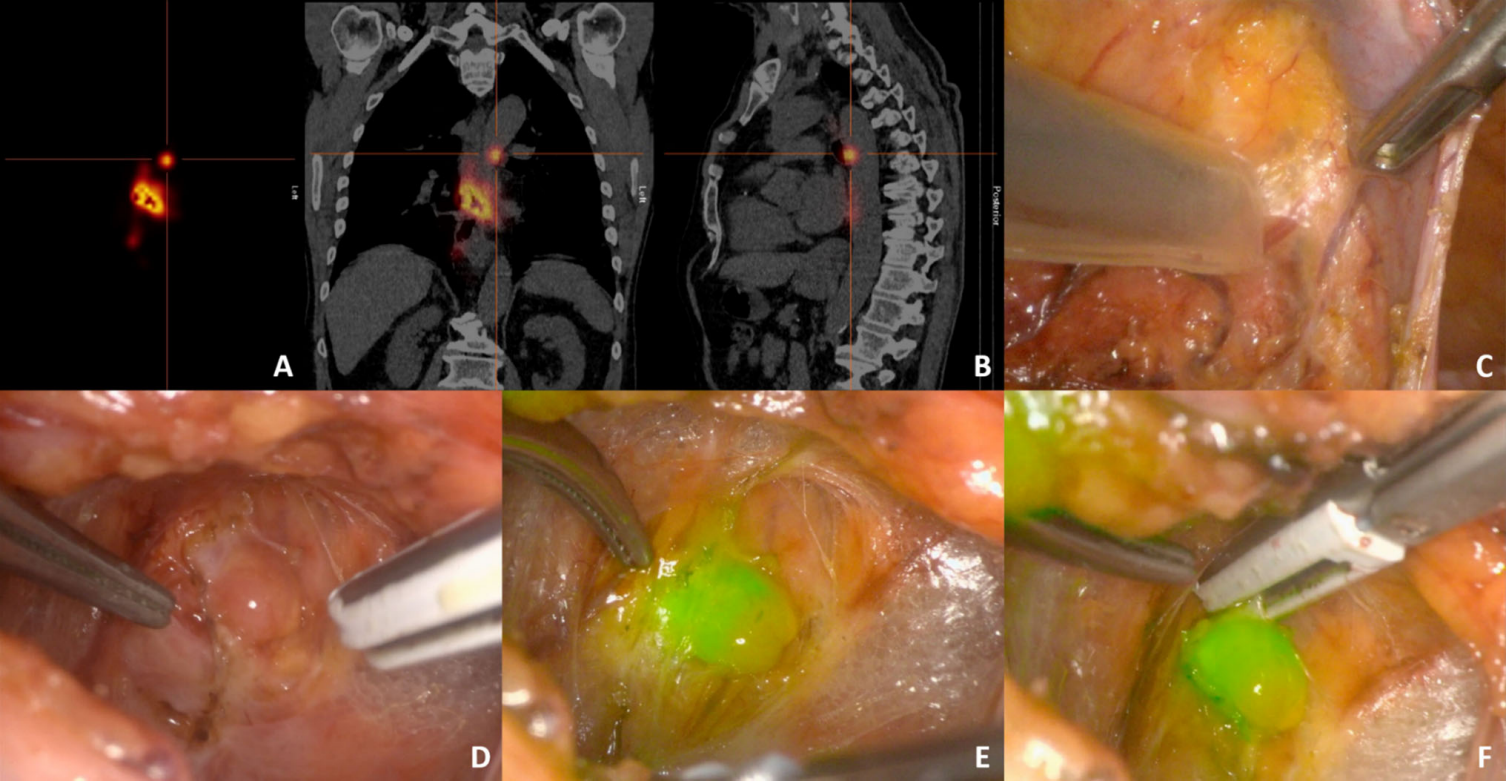
Identification of a sentinel node located at the aortopulmonary window. **A** Lymphoscintigraphy 2 h after endoscopic injection of the hybrid tracer showed the injection site and an intrathoracic sentinel node. **B** This was combined with a SPECT-CT to detect the sentinel node location. **C** The thoracolaparoscopic gammaprobe confirmed high radioactivity uptake during the thoracic phase of surgery, **D** after which the sentinel node could be identified. **E** The sentinel node was also clearly visualized as indocyanine green positive when the camera view was switched to near-infrared. **F** Subsequently, thoracoscopic resection of the sentinel node was performed under near-infrared vision

Although the SNNS procedure has been described previously,^16^ we report the method in detail since small adaptations have been made to the existing protocol. Patients were admitted to the hospital one day before surgery. The hybrid tracer ^99m^Tc-ICG-nanocolloid (2 cc, 100 MBq, 0.17 mg ICG, GE Healthcare, Chicago, Illinois, USA) was divided over four quadrants around the endoscopic resection scar by means of submucosal endoscopic injection (Supplementary Fig. 1). Subsequently, planar images with a gamma camera were conducted from the neck to the upper abdomen 2 h after injection of the tracer, directly followed by SPECT-CT (Fig. 1). These preoperative images were used to identify the anatomical location of all SNs, including distant SNs, and were evaluated by experienced nuclear medicine specialists (B.K. or R.B.). Minimally invasive SNNS was performed the next day according to the site’s standard of care, either by conventional thoracolaparoscopic or robotic-assisted minimally invasive surgery. SNs were detected during surgery with a thoracolaparoscopic gammaprobe (Europrobe 2/3, PI Medical Diagnostic Equipment B.V. Raamsdonksveer, The Netherlands) and an NIR camera [Firefly camera integrated in the da Vinci surgical system (Intuitive Surgical Inc., Sunnyvale, CA, USA) or Pinpoint fluorescence imaging system (Stryker, Kalamazoo, MI, USA)]. The SNNS procedure typically consisted of an abdominal and thoracic phase, with the order depending on the location of the SNs on preoperative imaging and the surgeon’s preference. Both phases started with SN identification using the gammaprobe (count rate of ≥ 10 times the measured background radioactivity in the operating room), followed by confirmation of ICG positivity with an NIR camera and eventual dissection of the SNs (Fig. 1; Supplementary Video 1). Radioactivity was confirmed ex vivo by repeating a gammaprobe measurement using a second, handheld gammaprobe. Additionally, the peritumoral region was carefully inspected with the NIR camera to localize SNs not visualized on preoperative imaging due to high tracer activity at the injection site. After resection of these ICG positive peritumoral lymph nodes, radioactive uptake was measured ex vivo using the handheld gammaprobe. After finalization of SN detection and resection, the thoracic or abdominal cavity was checked with the gammaprobe and NIR camera to confirm absence of remaining SNs. Lymph node stations were classified according to the 8th edition of the American Joint Committee on Cancer esophageal cancer staging system.^17^ Identified SNs were resected if located in locoregional lymph node stations 2, 4–8, 10, and 15–20, and additionally in the hepatoduodenal ligament.

### Histopathological Evaluation

All specimens were processed according to the current standard of care with fixation in formalin and embedding in paraffin. All SNs were cut at three levels, while other lymph nodes (non-SNs) were cut at one level. Subsequently, all slides were stained with hematoxylin and eosin for evaluation of metastases by an expert gastrointestinal pathologist (L.B. or S.M.). When no metastases were observed, additional immunohistochemical keratin staining (AE1-3) was performed on each level to detect possible micrometastasis.

### Evaluation of Esophageal and Gastric Function

Prior to SNNS, a high-resolution manometry and scintigraphic gastric emptying test for solid food were performed, according to the site’s standard protocol, to assess esophageal and gastric functioning. Both procedures were repeated 3 months postoperatively to detect potential functional disorders. In addition, an upper endoscopy was performed at 7 (±2) days after SNNS to evaluate the presence of ischemia in the esophagus and stomach.

### Follow-Up

Additional treatment was based on the histopathological assessment of the dissected SNs. In case of tumor-negative SNs, patients were monitored closely with stringent endoscopic follow-up. This follow-up consisted of upper endoscopy supplemented with endoscopic ultrasound every 3 months during the first 2 years after SNNS. Endoscopy was performed to survey any residual Barrett’s mucosa, which was generally kept under surveillance for at least 1 year after endoscopic resection, before any additional thermal ablation. Subsequently, endoscopic eradication treatment was initiated per discretion of the treating physician. Endoscopic ultrasound was performed to evaluate suspicious lymph nodes followed by fine needle aspiration when indicated. As an additional safety precaution, a PET–CT or CT thorax–abdomen was performed at 1 year after the initial endoscopic resection to evaluate the presence of distant metastases. In case of tumor-positive SNs, additional treatment was determined in a multidisciplinary meeting, taking the patient’s comorbidities, age, and preferences into account.

### Outcome Parameters

The primary endpoints were: (1) surgical morbidity, defined as any adverse event related to the SNNS procedure within 3 months follow-up, and (2) incidence of functional disorders, defined as postoperative impairment of esophageal and/or gastric functioning within 3 months follow-up. Secondary endpoints included: (1) percentage of patients with detectable SNs, either on preoperative imaging or during surgery, (2) number of SNs per patient, location documented, (3) concordance of preoperative imaging and intraoperative fluorescence- and gammaprobe-based SN detection, (4) number of resected (non-)SNs, (5) number of tumor-positive (non-)SNs, and (6) total procedure time.

### Statistics

No formal sample size was calculated considering this is a pilot study. A sample size of ten assessable patients was considered sufficient to evaluate feasibility and safety. Statistical analysis was performed using the Statistical Software Package IBM SPSS Statistics version 26.0.0.1 for Windows (SPSS, Chicago, Illinois, USA). No statistical comparisons were made, and only descriptive statistics, using medians with minimum and maximum values (range) or 25th and 75^th^ percentiles (p25–p75), were reported.

### Ethics

The study protocol and subsequent amendments were reviewed and approved by the Medical Research Ethics Committees United. Written informed consent was obtained from all study participants. A Data Safety Monitoring Board was established to monitor patient safety. This study was registered in the Netherlands Trial Register (NL8100).

## Results

### Study Population

Twelve patients with histopathologically confirmed high-risk T1 EAC and no evidence of lymph node or distant metastasis were enrolled between January 2020 and July 2022. Two patients were excluded prematurely due to a lack of operating room capacity during the COVID-19 pandemic (*n* = 1) and newly diagnosed comorbidity precluding surgery (*n* = 1). Ultimately, ten patients underwent SNNS per protocol of whom the results are reported hereafter (Supplementary Fig. 2). Baseline characteristics are summarized in Table 1. Histopathological evaluation of all endoscopic resection specimens revealed at least one high-risk feature in all patients (Table 2).

**Table 1.** Patient characteristics of the patients who underwent sentinel node-navigated surgery

		*n* = 10
Male sex, *n* (%)		10 (100)
Age in years, median (range)		69 (57–80)
Body mass index in kg/m^2^, median (range)		28 (23–36)
ASA classification, *n* (%)	*ASA I*	0 (0)
	*ASA II*	7 (70)
	*ASA III*	3 (30)
	*ASA IV*	0 (0)
Hiatal hernia in cm, median (range)		3 (2–5)
PPI 40 mg twice daily, *n* (%)		10 (100)
Prague classification in cm, median (range)	*Circumferential*	4 (0–10)
	*Maximum*	6 (1–10)
Endoscopic resection technique, *n* (%)	*EMR*	4 (40)
	*ESD*	6 (60)
Type of endoscopic resection, *n* (%)	*Piecemeal*	4 (40)
	*En bloc*	6 (60)
Adverse event during endoscopic resection, *n* (%)		0 (0)

*EMR* endoscopic mucosal resection, *ESD* endoscopic submucosal dissection, *PPI* proton pump inhibitor

**Table 2.** Tumor and histopathologic characteristics of the patients who underwent sentinel node-navigated surgery

Patient	1	2	3	4	5	6	7	8	9	10
*Tumor characteristics*										
Location in cm from bite block	30	32	33	37	33	40	34	35	38	35
Primary Paris component	0–IIa	0–Is	0–IIa	0–IIa	0–IIa	0–Ip	0–IIa	0–Is	0–IIa	0-IIa
Secondary Paris component	0–IIb	0–IIa	0–IIc	0–IIc	0–IIc	0–IIa	0–IIb	–	–	–
Length in mm	40	20	30	20	20	15	60	40	15	20
Circumferential extent in %	50	25	25	50	20	30	40	25	50	50
*Histopathologic characteristics*										
Histopathologic subgroup	T1b	T1a	T1b	T1b	T1a	T1b	T1a	T1b	T1b	T1b
Invasion depth	SM1	M3	SM3	SM2	M3	SM1	M3	SM3	SM2	SM1
Differentiation grade	Poor	Poor	Moderate	Poor	Moderate	Moderate	Poor	Moderate	Moderate	Moderate
Lymphovascular invasion	Yes	No	Yes	Yes	Yes	Yes	No	Yes	No	Yes

### Sentinel Node-Navigated Surgery

Selective lymphadenectomy using SNNS was performed a median of 115 days (range 71–173 days) after initial endoscopic resection. In all patients, endoscopic injection of the hybrid tracer was feasible with a median procedure time of 10 min (range 6–35 min). SNs were detected in all patients, both on preoperative imaging [median of 3 SNs (range 1–7) in median 3 lymph node stations (range 1–6)], and during surgery [median of 3 SNs (range 1–6) in median 2 lymph node stations (range 1–5)]. In five patients (50%), SNs were identified in both the thoracic and abdominal compartment on preoperative imaging. In the other five patients (50%), the SNs were located in a single compartment (thoracic compartment *n* = 3, abdominal compartment *n* = 2). In the patients with SNs located exclusively in the thoracic compartment, exploration of the abdominal compartment was omitted as determined in a multidisciplinary meeting. Three SNs, each in a different patient, were not approached at the surgeon’s discretion due to the location outside the standard surgical resection field [i.e., supraclavicular right *n* = 1, left thoracic compartment at the pulmonary ligament *n* = 1, and below the superior mesenteric artery *n* = 1 (Table 3)].

**Table 3:** Concordance of sentinel node detection on imaging and probe- and fluorescence-based detection per lymph node station

Patient		1^1^	2^1^	3	4	5	6	7^1^	8	9^2^	10
1	Supraclavicular										Imaging^3^
2R	Right upper paratracheal	Imaging, probe and ICG (1 SN)									
2L	Left upper paratracheal							Imaging, probe and ICG (1 SN)	Imaging, probe and ICG (1 SN)		
4R	Right lower paratracheal				Imaging, probe and ICG (1 SN)				Imaging, probe and ICG (1 SN)		
4L	Left lower paratracheal		Imaging, probe and ICG (1 SN)						Imaging, probe and ICG (1 SN)		
5	Aortopulmonary					Imaging, probe and ICG (1 SN)					
7	Subcarinal			Imaging, probe and ICG (1 SN)	Imaging, probe and ICG (1 SN)						
8M	Mid paraesophageal			Imaging, probe and ICG (1 SN)		Imaging, probe and ICG (1 SN)			Imaging, probe and ICG (2 SNs)	Imaging and probe (1 SN)	
8L	Low paraesophageal	ICG (1 SN)^4^		Imaging, probe and ICG (1 SN)			ICG (1 SN)^4^				ICG (1 SN)^4^
9	Pulmonary ligament	Imaging^3^									
15	Diaphragmatic		Imaging, probe and ICG (1 SN)		Imaging, probe and ICG (1 SN)						***Imaging, probe and ICG (2 SNs)***
16	Paracardial					Imaging, probe and ICG (1 SN)					
17	Left gastric			ICG (1 SN)^4^						Imaging	
19	Splenic artery									Imaging	
20	Celiac trunk			Imaging, probe and ICG (1 SN)			Imaging, probe and ICG (3 SNs)		***Imaging, probe and ICG (1 SN)***	Imaging and probe (1 SN)	
NA	Superior mesenteric artery								Imaging^3^		
Total number of resected SNs		2	2	5	3	3	4	1	6	2	3
Total number of tumor-positive SNs		0	0	0	0	0	0	0	1	0	1

In this table, boxes marked in bold italic refer to the location of the tumor-positive sentinel nodes in 2 of the 10 patients

*ICG* indocyanine green, *NA* not applicable, *SN* sentinel node

^1^Solely the thoracic compartment was explored due to the anatomical location of the SNs

^2^Dissection was considered incomplete after two SNs identified on preoperative imaging could not be detected intraoperatively

^3^These SNs were not resected due to the location outside of the standard surgical resection field

^4^These SNs were only detected with fluorescence-based imaging due to their proximity to the injection site with high background activity

Overall, the concordance between preoperative imaging and intraoperative SN detection was high (Table 3). In one patient (10%), the SN dissection was considered incomplete after two SNs could not be identified intraoperatively. This procedure was characterized by a lack of ICG fluorescence, which theoretically may be the result of suboptimal labeling to ^99m^Tc nanocolloid, causing rapid lymphatic clearance of ICG. All other SNs, identified on preoperative imaging and located within the surgical resection field, could be detected and subsequently resected during surgery. The median thoracolaparoscopic gammaprobe count rate was 185 counts per second (25p–75p, 128–432) in vivo compared with 535 counts per second (25p–75p, 343–1349) ex vivo after resection of the SN. All SNs identified intraoperatively with the gammaprobe were also detected as ICG positive with the NIR camera, except for two SNs in the patient with incomplete SN dissection. In four patients (40%) additional SNs located near the injection site were identified with the NIR camera. These SNs were not detected on preoperative imaging, or intraoperatively with the thoracolaparoscopic gammaprobe, as a result of the high background radioactivity of the injection site, but high radioactivity was confirmed ex vivo [median gammaprobe count rate 208 (range 123–720)]. After identification and resection of the SNs in each compartment, absence of in vivo radioactivity and fluorescence were confirmed, with the exception of the injection site. The total procedure time was median 125 min (range 46–213 min). No adverse events occurred during the SNNS procedures.

### Histopathological Evaluation

During histopathological evaluation, median 3 lymph nodes (range 2–14) were identified per patient in the resected SN stations. Per patient a median of 1 non-SNs (range 0–1) was resected, resulting in a total of median 4 lymph nodes (range 2–15) per patient. In eight patients (80%), none of the resected (non-)SNs showed signs of (micro)metastases. Based on the tumor-negative SNs, these patients were kept under strict endoscopic follow-up.

In the remaining two patients (20%), a metastasis was found in one of the resected SNs (Table 3). In one of these patients, the tumor-positive SN was located at the celiac trunk. This SN had a diameter of 4 mm on the preoperative scans and contained a metastasis of 2.2 mm, without extranodal extension (Supplementary Fig. 3). Restaging with upper endoscopy, endoscopic ultrasound and PET–CT revealed no intraluminal recurrence or additional metastases. Considering the patient’s older age and comorbidities, the patient was advised to undergo a watchful waiting approach consisting of strict endoscopic and radiologic follow-up, as determined in a multidisciplinary meeting. The other patient had a SN at the diaphragmatic crus, measuring 6 mm on preoperative imaging, which contained a micrometastasis of 1.5 mm (Supplementary Fig. 4). This patient also had a supraclavicular SN which was not resected due to the location outside the standard surgical resection field. This extraregional SN was subsequently evaluated with an ultrasound-guided cytologic fine needle aspiration showing no additional metastasis. After discussion in a multidisciplinary meeting, the patient preferred to undergo strict endoscopic and radiologic follow-up, balancing his relatively young age, the potential risks of an esophagectomy, and the presence of only a single micrometastasis.

### Follow-Up

Patients were hospitalized for a median of 2 days (range 1–3 days) after the SNNS procedure. None of the patients developed signs of esophageal or gastric ischemia. One patient (1/10, 10%) experienced an adverse event during the first 3 months of follow-up. This patient developed prolonged neuropathic pain as a result of the thoracoscopy, which improved after an intercostal nerve block. In all patients, the esophageal and gastric function were preserved postoperatively (Supplementary Table 1). All patients were alive after 3 months of follow-up, without evidence of any (new) lymph node or distant metastases. During the first endoscopic follow-up at 3 months, three patients were diagnosed with a metachronous lesion in the remaining Barrett’s segment. All lesions were amendable for endoscopic resection and showed a lower histopathology grade compared with baseline (low-risk T1a EAC *n* = 1, high-grade dysplasia *n* = 1, and low-grade dysplasia *n* = 1).

## Discussion

This study is the first to evaluate an esophageal preserving treatment algorithm for patients with a high-risk T1 EAC, consisting of a radical endoscopic resection of the tumor followed by SNNS with the hybrid tracer ^99m^Tc-ICG-nanocolloid. This minimally invasive treatment strategy appears to be feasible and safe without impairment of the esophageal and gastric function. In carefully selected patients, SNNS may therefore be a valuable instrument to limit lymphadenectomy, preserve the esophagus, and thus personalize additional treatment on the basis of lymph node involvement.

Nonetheless, SNNS for esophageal cancer can be challenging. The esophagus is surrounded by a complex, multidirectional lymphatic network resulting in a wide variation in SN locations.^13^ Precise injection of the tracer in four quadrants around the endoscopic resection scar is therefore crucial to cover all lymphatic ducts. In addition, the tracer needs to have long durability since endoscopic injection is performed the day before surgery. Lastly, planning and logistics are complex and demanding due to the short time frame in which a patient has to undergo multiple procedures. This may potentially complicate future implication in routine daily clinical practice.

Originally, high-risk T1 adenocarcinomas limited to the mucosa were excluded from the study protocol. However, this specific subcategory was added following a recent publication that demonstrated a higher rate of LNM (20%) than previously reported.^3^ In our cohort, 3/10 (30%) patients were diagnosed with a high-risk T1a cancer on the basis of either poor differentiation (*n* = 2) or presence of LVI (*n* = 1). While none of the resected SNs in these patients contained tumor cells, this subgroup is too small to compare with the available literature and draw firm conclusions.

In contrast, two of the remaining seven high-risk T1b cases were found to have a tumor-positive SN. When combining the current study with preceding SNNS studies from our research group, 3 out of 17 patients (18%) with high-risk T1b cancer had LNM.^15,16^ This rate is in line with recent retrospective series on the risk of LNM in high-risk tumors,^2,3,6^ and stresses the importance of tailored treatment. More importantly, these cases confirm that SNNS is able to detect tumor-positive lymph nodes as SNs, although the numbers are still limited. Interestingly, the presence of LVI was the common denominator in both cases with tumor-positive SNs.

Initially, patients with a tumor-positive SN were intended to undergo a subsequent esophagectomy with complete lymph node dissection. However, none of the two patients with a tumor-positive SN had any signs of residual disease upon restaging directly after SNNS. Therefore, both patients are currently being kept under strict endoscopic and radiologic surveillance, as determined in a multidisciplinary meeting, taking into account patient’s older age (*n* = 1) and presence of a single micrometastasis in combination with patient’s preference (*n* = 1). In these specific cases, one might debate if the outcome of the SN procedure did have any clinical consequences. On the other hand, if the tumor-positive SN was the only lymph node containing tumor cells, the selective lymphadenectomy may have been curative, and overtreatment may be prevented by provisionally restraining these patients from additional invasive treatment. Moreover, additional treatment can still be considered in case of any future new metastases during follow-up, although the lag time may influence oncological outcome and survival.

Strengths of this study are the multicenter setting in high-volume expert centers, with involvement of a consistent multidisciplinary research staff. Additionally, all study procedures were attended by a dedicated research fellow to ensure uniformity. Moreover, the combined tracer with long-lasting durability enabled precise injection during a single endoscopic procedure. Lastly, each endoscopic resection specimen was evaluated by an expert pathologist before patients diagnosed with a high-risk T1 EAC were enrolled.

This study also has several limitations that need to be addressed. Primarily, the sample size of ten patients was small, which was partly dependent on the rare patient category eligible for this pilot study. Future validation of the concept in a larger cohort with long-term follow-up will be challenging, especially considering the low incidence of LNM. In addition, as a direct consequence of the complex lymph node distribution around the esophagus, three SNs were located outside the standard surgical resection area and were therefore not approached. As discussed in a multidisciplinary meeting, these lymph nodes were not scheduled for a separate excision, but closely monitored during follow-up. Furthermore, in one patient two out of the four SNs detected on preoperative imaging could not be located during SNNS. Even though this procedure was complicated by the absence of intraoperative fluorescence, the other two SNs could still be identified with the gammaprobe solely. Lastly, the resolution of preoperative imaging was hampered by the high tracer activity residing at the injection site, which can conceal adjacent SNs. Even though the addition of ICG enabled detection of extra peritumoral SNs, the high tracer activity and fluorescence at the injection site may have impeded SN detection, particularly in patients with a large endoscopic resection scar. Therefore, other radioactive tracers with less shine-through effect might be interesting subjects for future research.^18^

Despite these promising short-term results of SNNS, the long-term follow-up of our cohort is required to determine the clinical value of this esophageal preserving strategy. In the future, SNNS might serve as an appealing treatment option between esophagectomy and a wait-and-see policy, which is currently being evaluated in an ongoing trial (clinicaltrials.gov, NCT03222635). Ideally, the new treatment algorithm with SNNS will be offered to a selected subgroup of patients with high-risk T1 EAC who are most likely to benefit from SNNS. Better risk stratification of these patients may be facilitated by further discriminating the absolute risk of LNM for each individual histopathological risk feature, in which the data from the registry on the watchful waiting approach may play a key role. On the other hand, SNNS might also be a valuable addition for those patients under endoscopic surveillance who develop LNM. In these selected cases, SNNS may enable selective resection of the tumor-positive lymph nodes, as well as identification of additional sentinel nodes at risk for metastases.

In conclusion, SNNS with ^99m^Tc-ICG-nanocolloid appears to be a feasible and safe instrument to tailor lymphadenectomy in patients with a high-risk T1 EAC, who underwent a prior radical endoscopic resection. The exact position of this new strategy in the treatment algorithm for high-risk T1 esophageal cancer needs to be studied in future research.

## Supplementary Information

Below is the link to the electronic supplementary material.

Supplementary file 1. Video of tailored lymphadenectomy using sentinel node navigated surgery

Supplementary file 2 (DOCX 9151 KB)
