# Supplementary material for: Feasibility and Safety of Tailored Lymphadenectomy Using Sentinel Node-Navigated Surgery in Patients with High-Risk T1 Esophageal Adenocarcinoma
Source: Ann Surg Oncol. 2023 Mar 23;30(7):4002–11. doi: 10.1245/s10434-023-13317-6 (PMC10035969; doi:10.1245/s10434-023-13317-6)
Supplement: Supplementary file 2 — (DOCX 9151 KB) [file 10434_2023_13317_MOESM2_ESM.docx]

**Supplementary Figure 1. Endoscopic injection of the tracer**

**A** This figure shows an endoscopic resection scar of 4cm in length between 12 and 4 o’clock, fully regenerated with neosquamous epithelium after a piecemeal multiband mucosectomy for a poorly differentiated high-risk T1a cancer with no lymphovascular invasion. **B** After endoscopic injection of saline in the submucosal layer of four quadrants around the resection scar, a 0.5cc of the hybrid tracer was injected in each saline collection (total 2cc).


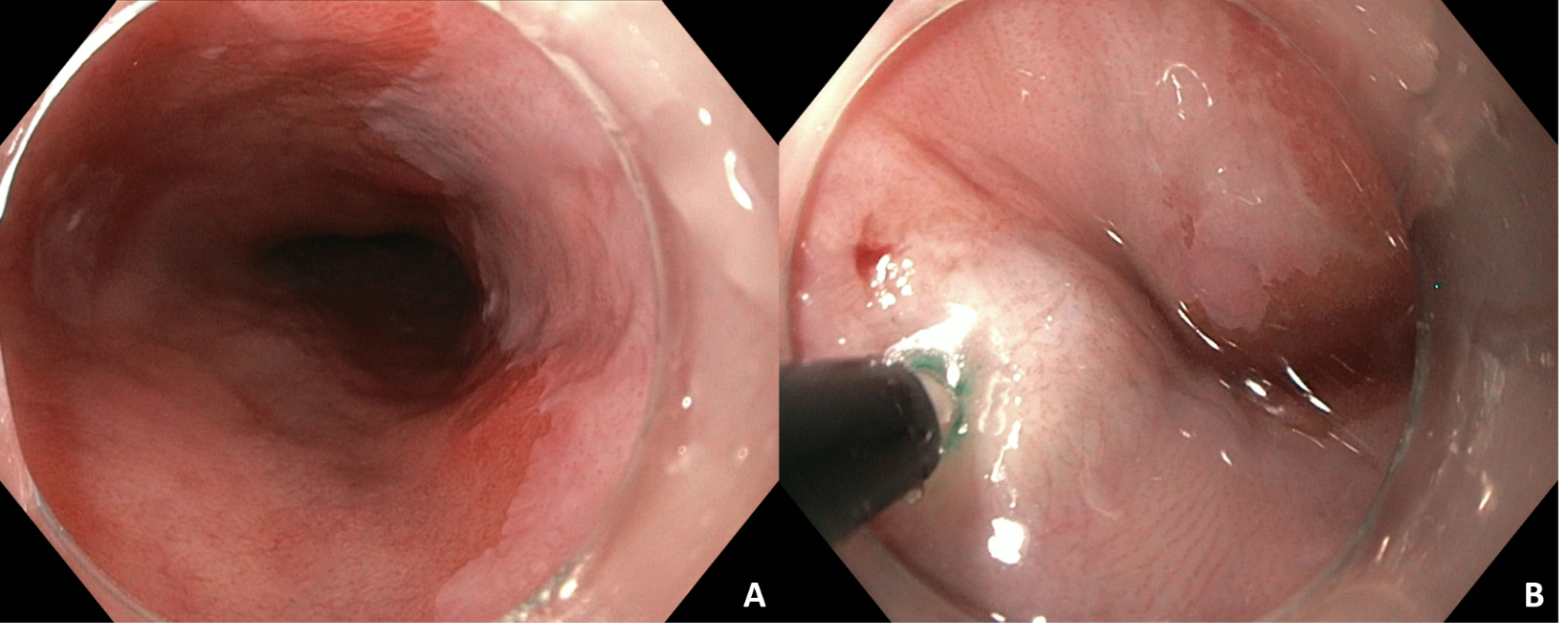


**Supplementary Figure 2. Patient flow chart**


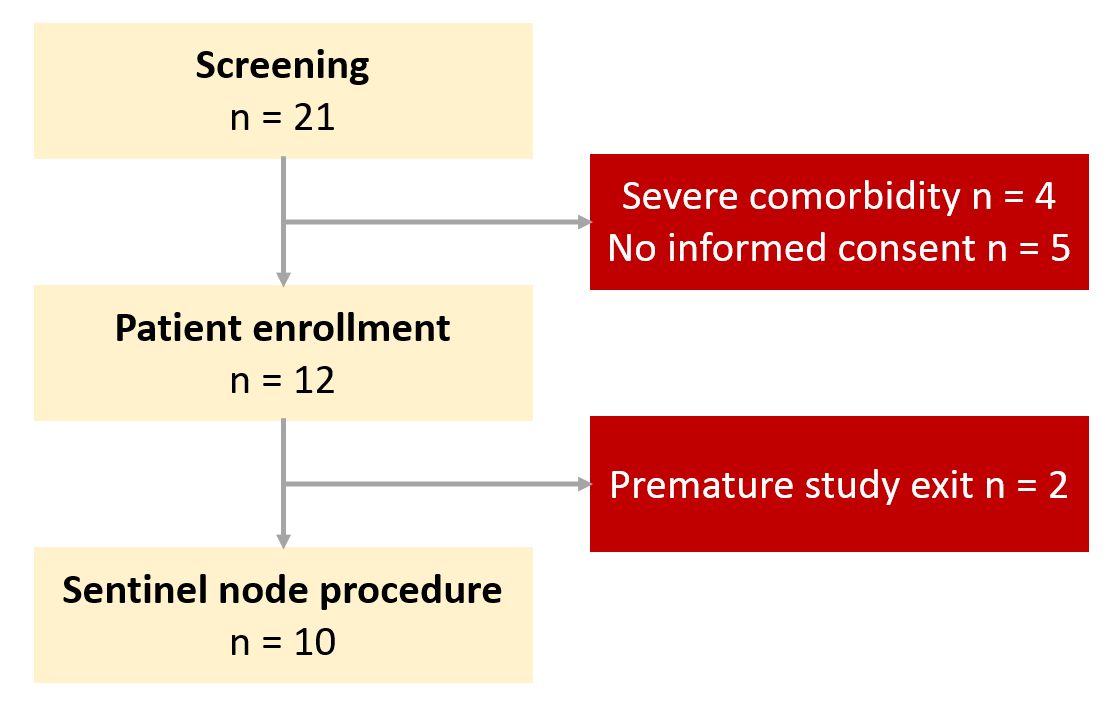
Twenty-one potential candidates were screened for study eligibility, of which 12 patients were enrolled. Ten patients eventually underwent sentinel node navigated surgery, after two patients were excluded prematurely due to a lack of operating room capacity during the COVID-19 pandemic (n=1) and newly diagnosed comorbidity precluding surgery (n=1).

**Supplementary Figure 3. Preoperative scans of patient with tumor positive sentinel node at celiac trunk**

**A** Lymphoscintigraphy showed the injection site of the hybrid tracer and multiple sentinel nodes in the thoracic and abdominal compartment. **B** As shown by the SPECT-CT, one sentinel node was located in the lymph node station at the celiac trunk. **C** The transverse plane of the SPECT-CT shows the near proximity of the sentinel node to the gastrohepatic ligament. **D** This sentinel node with a diameter of 4mm on the low-dose CT (*red circle*) contained a metastasis of 2.2mm.

**
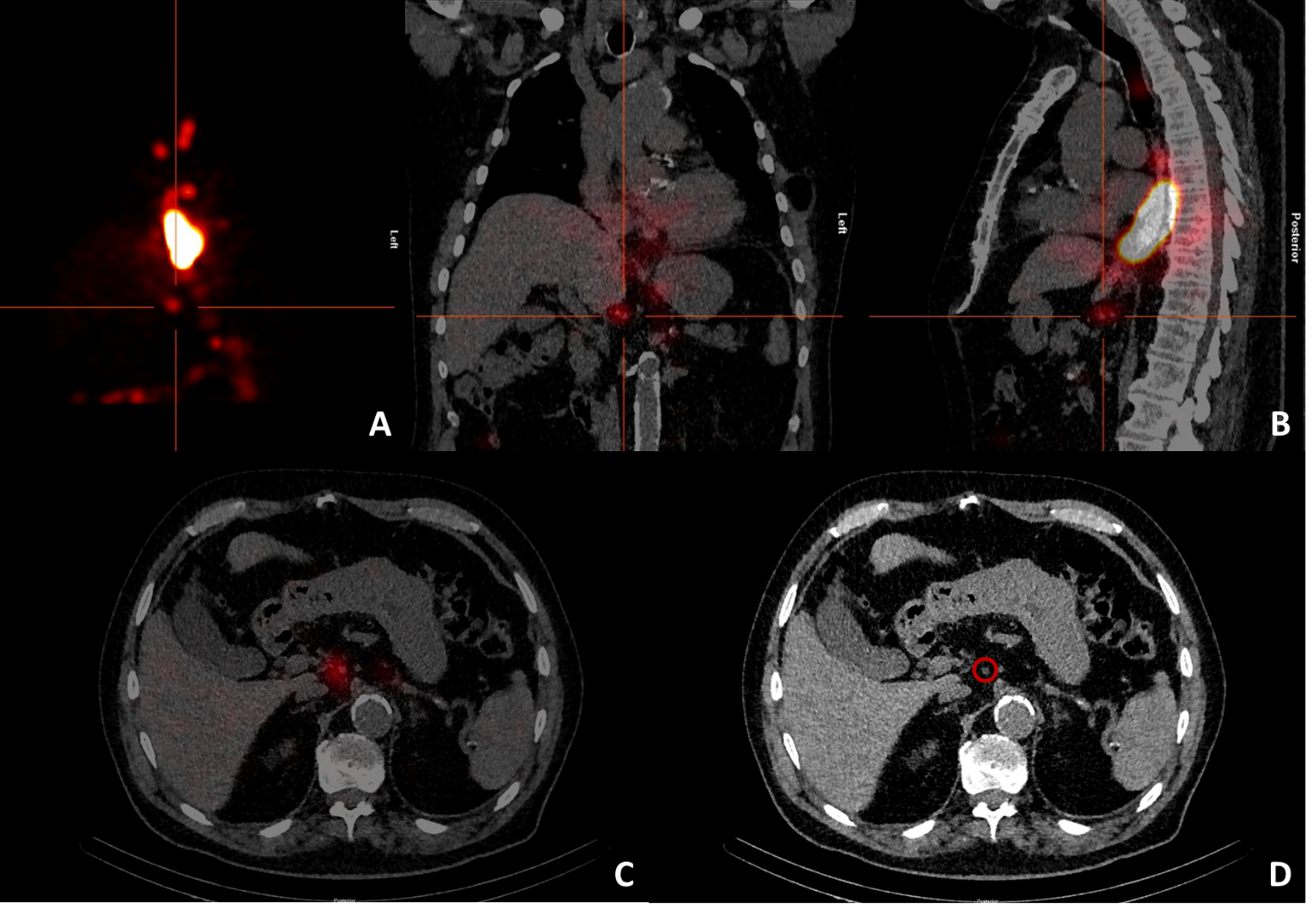
**

**Supplementary Figure 4. Preoperative scans of patient with tumor positive sentinel node at diaphragmatic crus**

**A** Lymphoscintigraphy showed the injection site of the hybrid tracer and a sentinel node in the abdominal compartment. **B** As shown by the SPECT-CT, this sentinel node was located in the lymph node station at the diaphragm. **C** The transverse plane of the SPECT-CT shows the near proximity of the sentinel node to the aorta. **D** This sentinel node with a diameter of 6mm on the low-dose CT (*red circle*) contained a micrometastasis of 1.5mm.

**
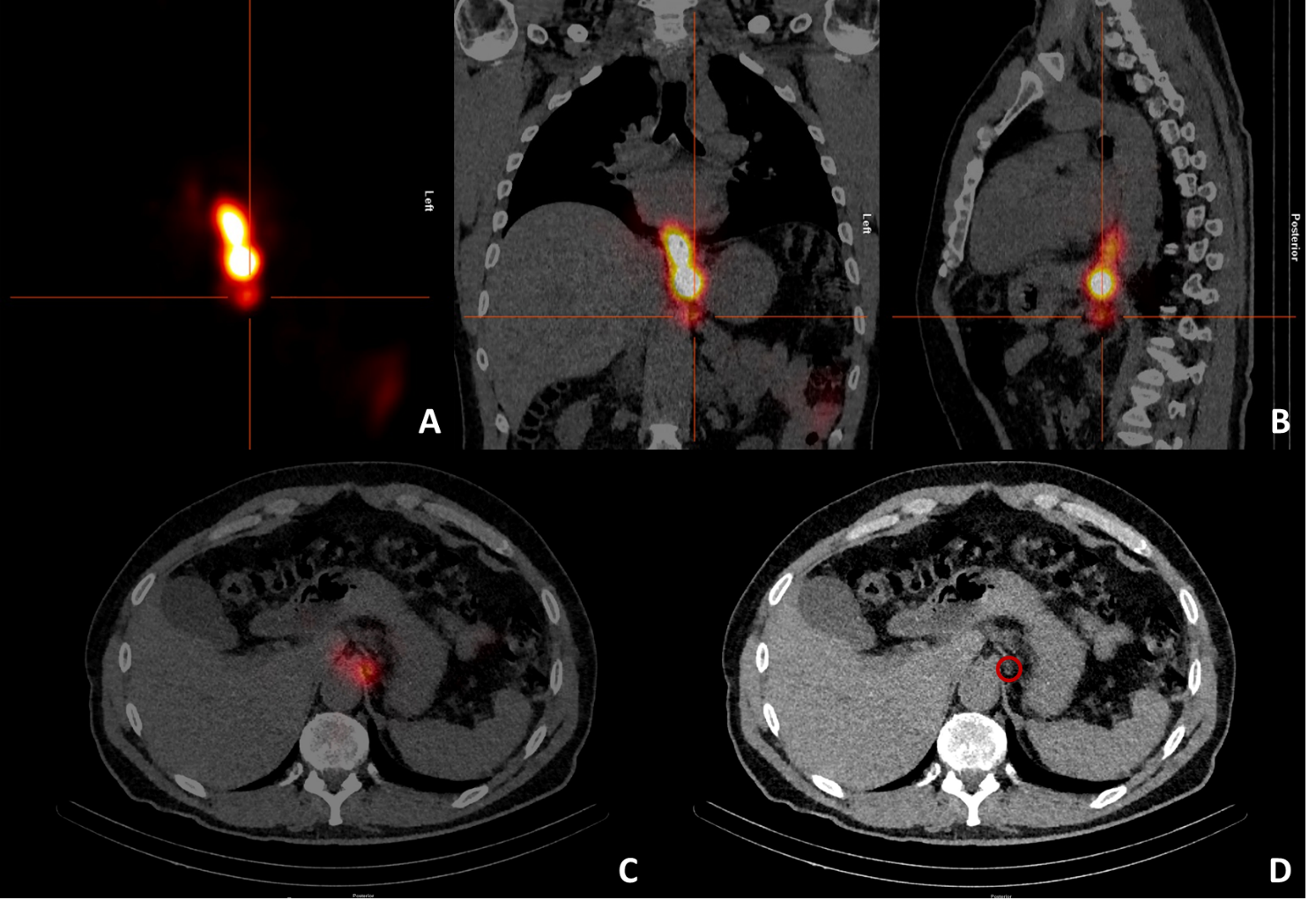
**

**Supplementary Table 1. Details of the pre- and postoperative esophageal and gastric function tests**

| **Patient** | **High-resolution manometry^1^** | | **Gastric emptying test^2^** | |
| --- | --- | --- | --- | --- |
|  | *Preoperative* | *Postoperative* | *Preoperative* | *Postoperative* |
| **1** | Absent contractility | Absent contractility | 35 | 38 |
| **2** | Ineffective esophageal motility | **'**Absent contractility | 15 | 35 |
| **3** | Ineffective esophageal motility | Ineffective esophageal motility | 26 | 12 |
| **4** | Ineffective esophageal motility | Ineffective esophageal motility | 10 | 33 |
| **5** | Absent contractility | Absent contractility | 3 | 4 |
| **6** | Absent contractility | Absent contractility | 14 | 24 |
| **7** | Ineffective esophageal motility | Ineffective esophageal motility | 38 | 47 |
| **8** | Ineffective esophageal motility | Ineffective esophageal motility | 9 | 9 |
| **9** | Normal | Normal | 36 | 3 |
| **10** | Normal | Normal | 7 | 16 |
| ^1^Diagnosis according to the Chicago classification.  ^2^Percentage retention after 2 hours (normal <45%). | | | | |
